# The main nursing metaparadigm concepts in human caring theory and Persian mysticism: a comparative study

**Published:** 2018-05-22

**Authors:** Lida Nikfarid, Nasrollah Hekmat, Arash Vedad, Anahita Rajabi

**Affiliations:** 1 *Assistant Professor, School of Nursing & Midwifery, Shahid Beheshti University of Medical Sciences, Tehran, Iran.*; 2 *Professor, Department of Philosophy, School of Literature and Human Sciences, Shahid Beheshti University, Tehran, Iran.*; 3 *BSc Candidate in Nursing, School of Nursing & Midwifery, Shahid Beheshti University of Medical Sciences, Tehran, Iran.*

**Keywords:** *Mysticism*, *Nursing theory*, *Human caring theory*

## Abstract

Metaparadigm concepts comprise the central issues in a discipline. Fawcett has named person, health, environment and nursing as the four main concepts of nursing that need to be comprehensively defined. The Human Caring Theory is significant because of its focus on the spiritual dimension of human beings. The aim of this study was to comparatively explain three of the main metaparadigm concepts of nursing in the Human Caring Theory and Persian mysticism, and find the similarities and differences that can help develop the theory and its application in societies with a theistic point of view. This comparative documentary study was done in two phases. First, a concept analysis was performed to find the attributes, antecedents and consequences of the concepts of human being, environment and health in the two fields of Persian mysticism and Jean Watson’s Human Caring Theory. Then they were apparently and deductively compared with each other. In spite of some similarities between the two perspectives, Persian mysticism was found to provide more comprehensive conceptualizations of the three main concepts of nursing.

## Introduction

Based on the literature, after experiencing the worldviews of *theism* and *naturalism*, modern nursing has adopted a kind of postmodern worldview: *a pantheistic monism* (1). In new nursing theories terms such as soul, energy, source and divine have been used in the three perspectives mentioned above, though with different meanings in each one (2). In the latter, they have found a more personal ontological place, which is related to creating one’s own reality (3). Most recent theories of nursing are affected by the philosophical ideas of existentialism and humanism, and Far East philosophies such as Taoism, Buddhism and Hinduism (1, 4), which are properly matched to the secular modern ideology of the West. As a result of the attempts to find a unique and distinct identity between other medical disciplines, the definitions of the main nursing concepts in these theories are holistic, subjective and transcendence-based (5). Jean Watson is a theorist in nursing who has persistently disseminated her “*Theory of Human Caring”* by publishing many articles and books, and providing lectures in professional seminars (6). She critiques the current trend of giving more attention to the expansion of solid and accurate knowledge in nursing through developing more specific theories. She cautions against the risk of ignoring the development of a theoretical basis for the main concepts in nursing, such as caring, which are needed for the humanistic face of the discipline (7). The Human Caring Theory has received attention because of the importance it places on the concept of caring as the essence of nursing (8). This theory has been developed over the past thirty years and is greatly influenced by theories of consciousness, existentialism, cognition sciences, quantum physics, interpersonal psychology, Taoism and feminism (9). The Human Caring Theory gives much importance to the spiritual, rather than the physical, dimension of human beings, and emphasizes the self-transcendence and self-actualization of the nurse in caring experiences. This is what makes this theory different from others that focus only on patients and do not notice spirituality as the preferable aspect (6, 9). In spite of the theorist’s attempts to provide a comprehensive worldview for nurse-patient encounters, there are some critiques on its theoretical philosophy and the definitions for its main concepts. The definitions of the concepts of person, health and environment are vague since they are abstract terms derived from multiple West and Far East philosophical thoughts (4, 6). Some concepts that are used in the definition of health and need to be briefly explained include: self-healing, harmony of mind, body and soul, and conciseness-transcendence (10). The concept of environment is defined as the internal and external factors effecting a person (8), which creates the need to answer questions about the existence of the world without considering the human factor. The indirect effects of global issues such as ethnic wars, terrorism and the ecological problems on every human being living in the world are ignored in this subject-based point of view. Affected by the thoughts of existentialism and humanism, the concepts of human being, nature, transcendence and even that energy-giving source (whether it is referred to as God, the divine power, or a similar title) are defined based on human-centrality. An evaluation of the current worldviews shows the need to seek another perspective on nursing, which may still be unknown in nursing literature. One possible perspective that has not been explored in nursing literature may be Persian mysticism. 

Mysticism is to reach the truth through intuition, revelation and alliance, which is an old creed in many cultures and nations (11). Despite some differences in the forms and manners, all types of mysticism have common points (12). According to Henry Corbin, an Islamist researcher (1978 - 1903), the non-secular Persian mysticism has a rich and comprehensive theoretical basis that is notable in the modern world (13). Alive, dynamic, and tremendously affected by Islam, Persian mysticism covers a range of worldwide themes and has the potential to be matched with many human science theories (14). Most nursing theories borrow their theoretical basis from other disciplines, and many nursing concepts are well conceptualized in Persian mysticism. To see the nursing concepts from a celestial mystical perspective is a new approach that can be used for the development of nursing grand theories. Any new approach in nursing should provide clear and precise definitions for the four nursing concepts of person (human being), environment, health and nursing. Jean Watson’s theory has a mystical nature; moreover, it is important to see things in the light of a recent and frequently studied theory. The aim of this study was to comparatively explain the concepts of person, health and environment in the Human Caring Theory and Persian mysticism. 

## Methods

In this comparative documentary analysis research, first we used the concept analysis method introduced by Walker and Avant (15) to identify, clarify and find definitions for three of the main concepts of nursing, that is, person, environment and health, in texts related to Persian mysticism and the Human Caring Theory. The Walker and Avant method consists of eight steps (Box 1), and was chosen as it is widely used and was highly related to the aim of the study. 

**Box 1:** Walker and Avant’s (2005) steps of concept analysis

**Steps**	**Actions**
1	Select a concept
2	Determine the purpose of the analysis
3	Identify all uses of the concept
4	Determine the defining attributes
5	Construct a model case
6	Identify antecedents
7	Identify consequences
8	Define empirical referents

The steps in the present study were: 1) determining the purpose of analysis, 2) finding all definitions and uses of the concepts, 3) identifying the characteristics or attributes, and 4) determining antecedents and consequences. For this aim, we searched related keywords in multiple steps in electronic databases such as Google, Google Scholar, Noor, SID, Magiran and PubMed, guided by experts in Persian mysticism. At the start, the first author read 2 textbooks and 3 articles on the history of Persian mysticism to achieve a general understanding of the subject. Next, papers on the works of Mulla Sadra, Avicenna, Suhrawardi, Attar and Rumi were examined due to the consensus of experts on their comprehensiveness and admissibility in explaining metaphysical concepts in a logical manner. At the concept analysis stage, 12 books, 48 articles and 4 theses were selected based on the creditability of the authors and journals. Qualitative content analysis was used to determine the attributes, antecedents and consequences of the concepts. Expert debriefing, long term involvement of the researcher with the subject, and concise reporting of the process of the study were used to increase the trustworthiness of the findings. Considering the aim of the study, we needed to find the similarities and differences between the attributes of the three concepts and provide a thick description for the philosophical foundations, so we did not construct any case models. 

In the second part of the study, we used a recommended method of comparison for religious and spiritual studies by Gharamaleki (16), and proceeded with the steps below:

1. Statement of the problem: This part focused on the question “What differences and similarities exist between the perspectives of Persian mysticism and the Human Caring Theory regarding the main concepts of nursing: person, health and environment?”
2. Limiting the scope of the comparison: In the present study, the ontological outlooks on the main concepts were explored in the two fields. Additionally, the attributes, antecedents and consequences driven for each of the concepts in the first part of the study were compared in both fields.
3. Providing a hypothesis: At this stage we formulated the hypothesis that “the theoretical foundations for the main concepts of nursing in the two fields of the Human Caring Theory and Persian mysticism have a lot in common, although the former could be more meaningful if explained from the non-secular perspective of Persian mysticism.
4. Listing differences and similarities: Here we found the differences and similarities between the concepts in both fields based on their obvious characteristics and what could be perceived thorough comparing appearances (Tables 1 - 3).

**Table 1:** Comparison between the concept of “person” in Persian mysticism and Human Caring Theory

	**Human Caring Theory**	**Persian Mysticism**
Antecedents		- Creation of the world and humans as a result of the love of the divine essence emerging
Attributes	A human being: - has dimensions of mind- body- Soul - is an existence on its way to becoming - is an embodied soul - deserves respect and is valuable - has authority to choose - can actualize his or her potentials	A human being: - has dimensions of body-soul - is an existence on its way to becoming - is an embodied soul - deserves respect and is valuable - has authority to choose - can actualize his or her potentials, which are the divine attributes - has an existential gradation - has certain faculties in each grade of existence - may evolve to the upper steps of gradation or not
Consequences		- Love of the divine essence is the motivation to transcendence

**Table 2:** Comparison between the concept of “environment” in Persian mysticism and Human Caring Theory

	**Human Caring Theory**	**Persian Mysticism**
Antecedents	- The Ten Caritas Processes can be used to provide a healing environment	- Creation of the world and the human being was the result of the love of the divine essence - Creation has gradations that end in the natural world (the environment of humans)
Attributes	- There are internal and external factors surrounding human beings There are factors that affect self-healing The human conscience can be connected to through caring experiences	- There are forces to help or inhibit the evolution of a Person - Pantheistic monism may be seen between creatures (as the unified essence and plural manifestations) - There is an active intelligence that can be connected to partial human intelligence - Creatures and meanings are various types of the divine essence attributes
Consequences	- Can be positive or negative for the harmony of mind-body-soul	- Creatures are interrelated for each other’s evolution - The formularized regulation governs the world

**Table 3:** Comparison between the concept of “health” in Persian mysticism and Human Caring Theory

	**Human Caring Theory**	**Persian Mysticism**
Antecedents	- The Caritas Processes	- Disease and suffering may be regarded as opportunities to develop the spiritual dimension (or not)
Attributes	- The harmony of mind-body-soul - A higher level of psycho-physical and social function, freedom from any form of disease, or an attempt to be from disease - The metaphysical potential for self- healing	- The harmony of soul and body to help one move upward in the spiritual dimension - To evolve through existential gradation - To know and move in step with the ethical, physical and social rules of the materialistic world
Consequences	- To self-heal through disease and suffering (or not)	- The ability to move to the upper grade (or not) - Emergence of attributes of the divine essence or exhibiting attributes pertaining to the lower grades

 Addressing real positions of differences and similarities: To do this, we used a systematic reasoning approach to provide a deep, meaningful explanation for the results. Considering the historical, theoretical and philosophical contexts and frameworks of each of the fields, we used a deductive approach to answer the central question posed in this study. 

5.     Explanation of the real positions of differences and similarities: Based on the ontological outlook derived from the literature and the experts’ standpoints, an explanation was provided for the results of the comparison between the concepts in each of the fields. 

## Results

In this section, the metaparadigms of nursing will be compared in the two fields of Human Caring Theory and Persian mysticism. The aim is to present a brief explanation for each metaparadigm in the two fields, and then proceed to provide a comparison. 

1) Person / Human Being

*A) The Human Caring Theory:*

According to Watson, a person has three dimensions of mind, body and soul, and is in fact an embodied soul on its way to becoming (17). Therefore, the spiritual dimension of a person has a higher value in the Human Caring Theory. A person is a being whose wholeness is valuable and deserves respect, assistance and care (4). From this perspective, soul is the higher sense of self and is similar to the psychological concept of self-actualization. Here, Watson’s existentialistic/humanistic ontological point of view shifts to a mystical one as she points out that a person can expand his or her inner healing power and reach an intuited mystic and even miracle-like experience through caring moments (4, 18). 

*B) Persian mysticism:*

**Figure 1:**
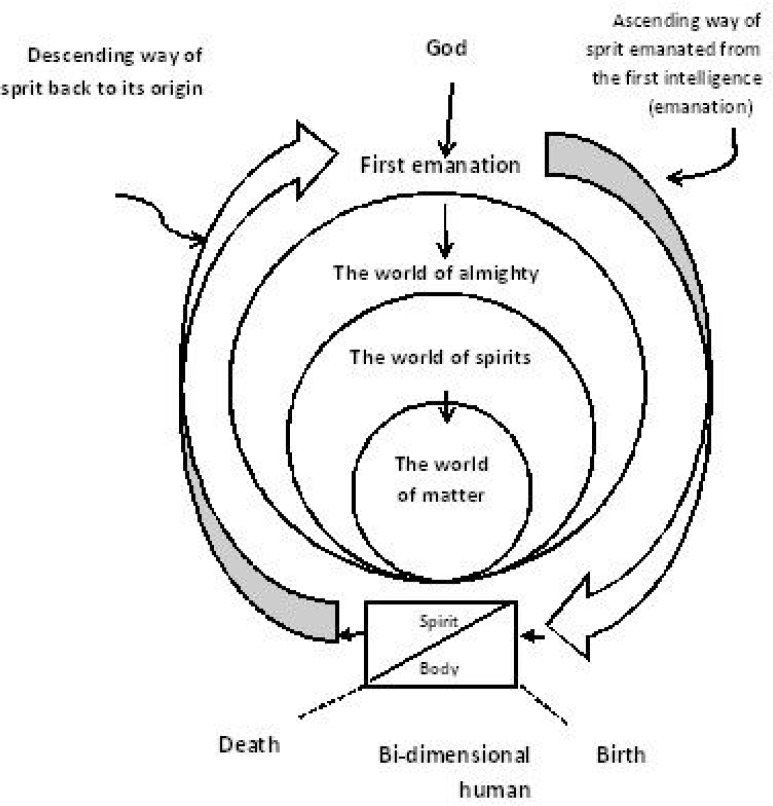
the diagram of gradations of the world of creation, and the way a spirit of a human from and to its origin (the first emanation) (figure is drawn by authors)

According to Persian mysticism, the world of creation began with the love of the divine essence (God) and emerged from two of His attributes: beauty and majesty. In Persian mysticism, a human being is a two-dimensional existence consisting of body and soul who is higher than all other creatures, being the only one who bears all the divine attributes and knowledge of the divine essence (God) as inherent potentials (19). The body is the lower dimension of the human existence: it has needs to serve, but will help the soul on the way to transcendence and actualization, that is, the emergence of the attributes of the divine essence through acts and thoughts (20). The human being is the only creature blessed with free will, empowered by intelligence and the faculty of speech. In addition, there is a gradation in existence with the sequence of solid-plant-animal-human, where each creature has the faculties to evolve and rise to the next grade (Figure 1). 

The ultimate perfection of a human being is to actualize the potentials of his or her grade (21). Humans can choose to ascend through their existential gradation toward being perfect human beings and gain more divine attributes, or stay in the lower grades and show attributes of those who are not evolved, for instance violence against others (an extreme manifestation of wrath, which belongs in the animal grade of humanity) (19). Intrinsic divine attributes such as mercy and grace inspire a kind of love for perfection in a human being, and are nurtured through everyday life practices such as caring, which can lead to self-actualization and self-healing (21). 

Rumi says:

*"Half made of water and clay*

*Half soul and half solar ray*

*Half on the shallow beaches lay*

*Half from the oyster's pearly play" *(22).

*C) Comparing the concept of person in the two fields (* Table *1):*

Watson’s perception of the human being as an embodied soul, whose spiritual dimension is more significant, is close to the standpoint of Persian mysticism. Nevertheless, the philosophical foundation of her theory is derived from an existentialistic/humanistic phenomenology, which is the center of everything in human beings (4). In this theory, the growth of a person and the actualization of his or her potentials are mentioned, but these potentials need to be well defined. From the celestial standpoint of Persian mysticism, affected by religious thoughts, God is the center of everything and the evolution of human beings is measured based on the extent of actualization of their divine attributes, which are their inherent potentials. A person is valuable in the Human Caring Theory, but the reason for this value is unclear. Additionally, it is not understandable why a person should be respected and valued when his or her acts are far from what is called humanity. In Persian mysticism, the faculties of each grade of humanity act for the maturity of that grade: from the time one is like a plant in the embryonic stage, or an animal before gaining the whole power of reasoning at the age of adolescence, until they gradually move far from their physical dimension to leave it at the age of seniority. Watson propounds the high value and spirituality of human beings (23), which is compatible with Islamic Persian mysticism. In Persian mystical thought, human beings are referred to as “Caliphs of Allah” (24), since they bear all the divine attributes as potentials. These attributes bear divine names, are the whole knowledge of the truth, and can emerge through willingly chosen, everyday human acts. According to Watson, there are possibilities to grow and excel. Nevertheless, from such a standpoint, the human being should be seen as a bi-dimensional existence that can move forward and backward in gradation, since an endless movement toward an unknown ultimate goal, like what is drawn in existential-based philosophies, does not make sense. In the Human Caring Theory, a source or God is inevitable, and is the empowering agent for human beings. It is introduced as a kind of metaphysical connection that can happen to a person. Thus, a mystical description of the human being can support nursing theories such as the Human Caring Theory.

2) Environment 

*A) The Human Caring Theory:*

In Watson’s Theory, there is great emphasis on a caring and healing environment, which can be provided by a nurse according to the existing literature. The internal and external factors that can help a person actualize his or her inner power of self-healing are called the environment. A nurse is considered an external factor that can offer assistance and care to a person through the Ten Caritas Processes including love, empathy, trust and teaching/learning experiences. Thus, a unity of the nurse and that person and self-transcendence of both will happen in such enchanted moments. It is notable that Watson only describes a caring environment and how it is provided by a nurse, and does not elaborate on the surroundings of a person in general in her theory. 

*B) Persian mysticism:*

**Figure 2:**
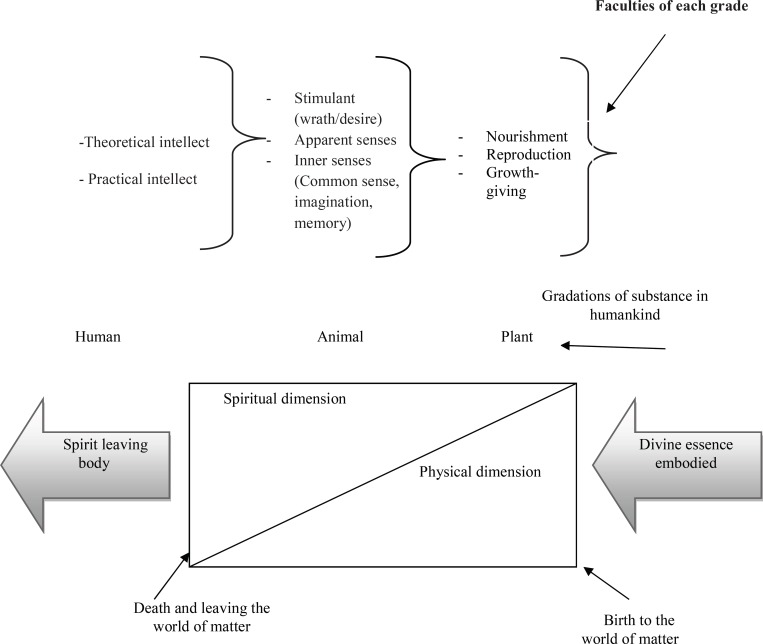
Diagram illustrating the bi-dimensional humankind, the substantial gradation and faculties of each grade in the world of matter, according to Persian mysticism (figure drawn by the authors)

From the perspective of Persian mysticism, the world and everything around a person are manifestations of God. The ultimate goal of creation is the emergence of divine attributes in humans through perfection of each level of their existential gradation, so the external and internal factors can be either inhibitors or facilitators in the process. In the natural world where physical, social and ethical issues are governed by formalized rules, people need to be in harmony with the ultimate goal of creation, or suffer. According to Persian mysticism, there is gradation for the worlds of existence as well, which are in agreement with the various levels of human perception (Figure 2). 

*C)* *Comparing the concept of environment in the two fields (* Table 2 *):*

It seems that both perspectives are mindful of how environmental factors may affect the evolution of a person positively or negatively. It is understandable that a nursing theory should acquire a practical point of view, but considering the complicated and challenging modern societies, there is need for a more holistic worldview. From a mystical perspective, the environment is more than just what surrounds us. In fact, man is living alongside an enormous set of material and nonmaterial creatures, is whole but has a unified essence, is one of the manifestations of God, and is moving toward a determined aim. Human beings are the only creatures who are free to choose the way to perfection or stay away from it. Human beings and all other creatures make a unit where all are interrelated to one another, although they may belong to different grades of existence, and the acts of everyone affect this unified whole, themselves and others.

3) Health 

*A) The Human Caring Theory:*

Watson defines health as harmony of the body, mind and soul; as a high level of physical, mental and social performance; and absence of or an attempt to eliminate diseases (9). Healing means regaining wholeness, which is a new, different and better state than the one in which the patient was previously. It is moving toward transcendence, as well as physical, mental and spiritual wellness. Healing is defined as finding a new meaning for the disease and the recent condition (25). Identifying healing as a spiritual function, Watson believes in human beings’ potential metaphysical ability to self-heal and achieve supreme consciousness (26). She also supports a unique way of making meanings for illness/wellness experiences by each person, and the possibility of perceiving these meanings during the moments of care by the nurse (27, 28).

*B) Persian mysticism:*

Although the spiritual dimension is preferred and its perfection is the aim of the creation of human beings, spirit is confined by the physical dimension in material life. As the spirit does not belong to this structure and is a divine essence, living in the natural world and enduring its attributes cause pain and suffering. Over the centuries human beings have unsuccessfully tried to conquer everything that made them suffer. Furthermore, if the soul begins to move away from its earthly dimension, it can find a way to join the divine spirit and achieve peace or what is considered a healthy state in mysticism. Therefore, human beings choose to move downward or upward in this continuum of soul-body dimensions. The farther one moves from the end pertaining to the soul, the more suffering one will experience. 

*C) Comparing the concept of health in the two fields (* Table 3 *):*

 Watson sees health from a phenomenological perspective, in terms of what it means to people in their illness-wellness experiences, and a state that should be improved in caring moments. Like other cognitivists, she believes the human being is an existence that gives meaning to concepts and entities, and one that needs to rise to perfection through life events. During caring moments, the Caritas Processes help nurses improve the mentality of patients toward their illness. Although according to Watson a caring moment can lead to a spiritual/mystical experience for both the care-giver and the care-provider, the philosophical stances do not give any additional information about it. Health is a spiritual concept in the Persian mystical perspective too. It is regarded as an intuitive movement toward perfection, i.e., the emergence of divine attributes, in spite of limitations that are of a sensual nature. Thus, health is possible to achieve even in the presence of disease, and may even be considered as an opportunity for spiritual growth (29).

*"Hafiz do not complain*

*For union try*

*Put up with the pain*

*Whenever it comes by"* (30).

Nevertheless, both of these perspectives are similar in terms of addressing the spiritual dimension and potential of individuals for healing by the use of their metaphysical power. One approach to treating many physical and psychological problems in modern psychology is changing perceptions. While this viewpoint does not specify what humans should think about incurable diseases and pains in order to reach a state of transcendence and well-being, in Persian mysticism it is recommended to view them as the mercy of God, as a way to begin to move toward spirituality and feel closer to God (which Watson calls *the source*). Watson interestingly applies a mystical meaning to the spiritual linkage of two persons (a nurse and a patient) engaging in a caring experience. In an Islamic mystical context, this experience serves to help both oneself and other people acquire more divine attributes. Healing happens when a new meaning of life and its events are gained through interpreting pain as an opportunity for growth and getting closer to the spiritual dimension. While one of the critiques of the Human Caring Theory is that it cannot explain the relationship between the physical and spiritual aspects of human nature, Persian mysticism has rich theoretical bases that can help interpret the role of the physical dimension. Persian mysticism considers the human being as a gradational existence with certain faculties for each grade, and proposes to keep equilibrium in the function of each faculty to gain more health. For instance when a person in the plant grade has problems in the faculty of nourishment, this can lead to health issues like obesity. 

## Conclusion

Nursing is a caring profession, and since care is defined as the process of helping others grow, it is necessary to provide a theoretical basis that can demonstrate how to help all the bio-, psycho-, socio- and spiritual potentials of a human grow. As a preliminary phase to find a more comprehensive theory for nursing, we compared one of the current theories with a theistic point of view. Our findings showed some common points between definitions for the main concepts of nursing in the Human Caring Theory and Persian mysticism, even though the former has a human-centered structure, and the latter a God-centered one. Through this comparison, one can find the main similarities and differences between the two perspectives toward the main central concepts of nursing. Nursing has begun to experience conceptualization in a way as to examine concepts from a metaphysical perspective. The lack of empirical support is a limitation, but it seems that for a holistic standpoint it is impossible to set aside the person-centered lens.
